# Annotations

**Published:** 1909-01-23

**Authors:** 


### ANNOTATIONS.

### A Munificent Endowment of Medical Research.

The Medical School of the London Hospital has recently been the recipient of a benefaction, the provisions of which are in many respects unprecedented in this country. A sum of £20,000 has been placed in the hands of its trustees, who undertake to invest the money to the best advantage and to pay the income yearly into the hands of three administrators selected by the donor, who are to spend it in the advancement of medical research and the promotion of higher education in medicine. The administrators are the Chairman (Mr. Sydney Holland) and two members of the acting staff of the hospital. It has been settled that this money is to be spent on increasing the facilities for research, and not for the routine teaching of candidates for examination. The benefits derived from the gift will not be confined to those students educated at the London Hospital, but will be open to qualified medical men from any part of the British Empire who are willing to give up their time to advancing medical knowledge within the walls of the London Hospital or College. It is undoubtedly true that young men capable of doing good scientific and research work are often forced too soon into the routine of practice or teaching by the necessity of having to earn their livelihood. This fund will enable its administrators to free a certain number of carefully-selected men from this pecuniary anxiety, and thus enable them to apply themselves with whole-hearted devotion to any researches they may select. Even a small band of enthusiastic workers not only furthers knowledge but creates an atmosphere which favours fertility of thought and freshness of idea. The donor of this munificent gift wishes to remain anonymous, in the hope that the fund which he has thus started will be added to by others, and that in time it may become of such magnitude as to be of great use to the present and to all future generations in the fight against and the prevention of disease.

### Science and the Arts.

Sir Clifford Allbutt's presidential address last week to the Association of Public School Science Masters, on the function of science in education, contained some of those fine thoughts draped in distinguished language which we have come to expect from him. The following passage, for instance, is in happy contrast to the platitudes which are sometimes brought out on similar occasions: "As in their date and degree all human things fade, science is the organ by which we recover from them the principles of strength and beauty, and learn to adapt the newly won principles to new creations." To the denial of creative power itself to science, however, in which Sir Clifford Allbutt thinks the humanist justified, many will object. Of what other nature was the faculty by which Newton "conceived in his mind before the completion of his twenty-fourth year the three grand discoveries which formed the glory of his life"—the theories of gravitation, fluxions, and light? The reconstructive mind of the great scientist differs, of course, from the constructive mind of the great artist, but the first, no less certainly than the second, gives to the world something which is new to it. As Venn has well insisted, in any original investigation there is at first a stroke of insight or creative genius demanded: most probably in nine cases out of ten the investigator himself supplies this. It is often loosely said that poets and artists give men of science ideas to work on, but the whole history of the growth of knowledge shows that the number of those who have done so with any success is exceedingly limited. After Pascal and Goethe names are hard to find.

### Consumption and Psychology.

In this happy country written medical controversies are relegated to the correspondence columns, where their lively career is interrupted in due course by an editorial closure. The patient German, on the other hand, often makes of them a series of articles —the headings of which may sound antiphonally through quite a number of issues. Of the latter plan there is an example in Vols. VIII. and IX. of the "Beiträge zur Klinik der Tuberkulose." A Dr. Köhler writes to begin with on disturbances of psycho-physical equilibrium in consumptives, taking up the seemingly reasonable position that these may be either acquired and tuberculo-toxic, or else dependent upon hereditary cerebral abnormality. To him, in the language of the stage, enters (in Part IV.) Dr. Krämer, who will have none of these explanations, but considers such a well-known thing, for example, as the *spes phthisica*, simply the result of auto-suggestion leading its subject to disregard unpleasant prospects. Dr. Köhler returns to the charge, and in a paper with a title running to some forty words, beginning "Zur psychologischen Analyse in der Medizin . . ." answers his critic's objections and re-establishes himself. Dr. Krämer we shall no doubt hear again from later. That the consumptive has peculiar psychological as well as physical traits is no new statement. Letulle's characterisation is that of a person with a tendency to form vast plans, and to show feverish energy in carrying them out. A well-known British psychologist corroborates this fairly well. Havelock Ellis, too, tries to show that the work of consumptive geniuses, such as Keats, Chopin, Watteau, Stevenson, and Priestley, has certain broad characteristics. This is a class of investigation with which French medicine busies itself a good deal. Though somewhat conjectural, it perhaps shows that if such intensive study as, with the stimulus of therapeutic expectation, has been given to the "seed" of tubercle, were but devoted to its "soil," certain statements now made by pronounced "contagionists" might come to appear as the results of a bacteriological craze.

